# Prior tonsillectomy is associated with an increased risk of esophageal adenocarcinoma

**DOI:** 10.1371/journal.pone.0235906

**Published:** 2020-07-22

**Authors:** Katherine S. Garman, Teminioluwa A. Ajayi, Harold J. Boutte, Shih-Ting Chiu, Richard J. von Furstenberg, Benjamin R. Lloyd, Cecelia Zhang, Mark W. Onaitis, Shein-Chung Chow, Shannon J. McCall

**Affiliations:** 1 Division of Gastroenterology, Department of Medicine, Duke University, Durham, North Carolina, United States of America; 2 Department of Medicine, Vanderbilt University Medical Center, Nashville, Tennessee, United States of America; 3 Division of Gastroenterology, Department of Medicine, Northwestern Medicine, Chicago, Illinois, United States of America; 4 Providence Health and Services, Portland, Oregon, United States of America; 5 Division of Cardiovascular and Thoracic Surgery, Department of Surgery, University of California San Diego, La Jolla, California, United States of America; 6 Department of Biostatistics and Bioinformatics, Duke University, Durham, North Carolina, United States of America; 7 Department of Pathology, Duke University, Durham, North Carolina, United States of America; Baylor College of Medicine, UNITED STATES

## Abstract

**Background:**

Esophageal cancer is a deadly cancer with 5-year survival <20%. Although multiple risk factors for esophageal adenocarcinoma (EAC) including obesity, GERD and smoking have been identified, these risk factors do not fully explain the rising incidence of EAC. In this study, we evaluated the association between prior history of tonsillectomy and EAC. Our goal was to determine whether tonsillectomies were more frequent in patients with EAC (cases) than in our thoracic surgery controls.

**Methods:**

Cases included 452 esophagectomy cases, including 396 with EAC and 56 who underwent esophagectomy for Barrett’s esophagus (BE) with high grade dysplasia (HGD). 1,102 thoracic surgery patients with surgical indications other than dysplastic BE or esophageal cancer represented the controls for our analysis. The association of tonsillectomy and HGD/EAC were primarily evaluated by using univariate tests and then verified by logistic regression analysis. Baseline demographics, medical history, and thoracic surgery controls were compared by using χ^2^ tests or 95% CIs. Significant risk factors were considered as covariates in the multivariate models while evaluating the association between tonsillectomy and HGD/EAC. P-values or odds ratios were estimated with 95% confidence limits to identify significances which was more appropriate.

**Results:**

Tonsillectomy was more common in cases than controls and was found to have a significant association with esophageal cancer (19.9% vs. 12.7%; p-value = 0.0003). This significant association persisted after controlling for other known risk factors/covariates.

**Conclusion:**

A prior history of tonsillectomy was significantly associated with HGD/EAC and may represent an independent risk factor for the development of EAC. However, the underlying biology driving this association remains unclear.

## Introduction

Esophageal cancer remains a deadly cancer with a 5-year survival <20% [1]. While traditionally esophageal squamous cell carcinoma (ESCC) and esophageal adenocarcinoma (EAC) have been grouped together, recent genomic data demonstrate that these are two distinct cancer types [2]. Gastroesophageal reflux disease (GERD) and obesity and tobacco use are major risk factors for EAC [1, 3]. Screening and surveillance are performed for Barrett’s Esophagus (BE), the only known precursor lesion for EAC. However, given that EAC only occurs in a small percentage of patients with BE, identification of individuals at higher risk for disease remains a challenge [4]. An incomplete understanding of factors that increase risk for EAC continues to hamper efforts to develop meaningful prevention and treatment measures that reduce mortality.

Esophageal carcinoma affects over 450 000 people worldwide and the EAC subtype remains one of the few cancers with steadily increasing incidence and death rates [5, 6]. The incidence of EAC in the United States increased six-fold from 1975 to 2001, representing a relatively greater increase than other cancers including breast and prostate [7]. Pohl *et al*. concluded that this rise represents a true increase in burden of disease, perhaps due to a novel risk factor, rather than a consequence of changes in classification or diagnostic criteria [7]. Although increasing obesity rates and smoking have been identified as age-dependent risk modifiers, novel exposures or risk factors to explain this dramatic increase in incidence are yet to be identified [3].

A tonsillectomy, which involves removal of the tonsil or tonsillar capsule, is a common surgical procedure with over 500 000 procedures performed annually on children younger than 15 in the United States [8]. This procedure, reported as the most frequently performed surgical procedure in the early 20^th^ century, became less common in the late 1970s with increased use of antibiotics [8]. This declining trend in tonsillectomy has reversed as indications for tonsillectomy have shifted from infectious indications to sleep disordered breathing, which now represents the most common indication for tonsillectomy [8]. The tonsillectomy procedure, formerly thought to be benign, has been associated with increased risk for adult diseases [9–11], perhaps due to subsequent dysregulation of immune mechanisms. Tonsils are essential immune organs that protect the body from incoming bacteria and other pathogens. Tonsillectomies may cause changes to cellular and humoral immune function including changes to immunoglobulin production; while these immunologic changes have not been clinically significant in the pediatric population [12–14], more recent studies have shown that tonsillectomy may increase long-term risk for deep neck infection and malignancy [9, 10].

In our study, we used a case-control study design for an initial exploration of the association between the increase in tonsillectomies after the 1920’s and HGD/EAC using thoracic surgical cases and controls.

## Methods

The study was approved by the Duke Institutional Review Board (IRB Protocol Pro00039682) on 07/31/2012 with a waiver of informed consent. Consent was waived because the study involved secondary analysis of existing data and involved no more than minimal risk to the subjects. The study was performed in accordance with the Declaration of Helsinki, Good Clinical Practice, and applicable regulatory requirements.

### Study population

We recruited cases from a database of 603 patients who underwent esophagectomy at our institution between the years 2000 and 2010. Of the 603 patients, 452 represent the cases in our analysis, including patients who had EAC of the esophagus or gastroesophageal junction (GEJ) (n = 396) as well as patients who underwent esophagectomy for BE with high grade dysplasia (HGD) (n = 56). The esophagectomy cohort also included 81 patients with ESCC, but these were excluded from the primary analysis.

A control cohort was created from patients who had undergone surgical thoracic procedures. We identified 65 control patients from the esophagectomy cohort with benign esophageal disease (n = 46), other tumor such as a metastatic lesion (n = 11) or other cause (n = 8). We also recruited an additional 1037 thoracic controls from a large registry of 4750 thoracic surgery patients who underwent thoracic procedures other than esophagectomy. In selecting these additional thoracic surgery controls, patients with esophagectomy or HGD/EAC or a prior diagnosis of esophageal cancer were excluded. The rationale for this approach was based upon similar exposure to a thorough surgical and anesthesia history, including proper documentation of a surgical procedure like tonsillectomy. The initial sorting process excluded duplicate patients and generated 2775 unique potential controls. Patient inclusion for the control group were considered in conjunction with the statisticians on our study team and was based upon distribution of age and gender i.e. including patients within the interquartile range of ages stratified by gender. A chart-review was performed that excluded any other patients from the additional thoracic surgery controls with known history of esophageal cancer or esophagectomy; this resulted in exclusion of additional patients and affected the age and sex distribution of the control group. Those demographic factors were accounted for in the adjusted analysis. Indications for thoracic procedures were benign lung disease, mediastinal disease, pleural disease or infection, and “other”. The total control group for analysis included 1102 thoracic surgical patients.

### Chart review

We conducted an Electronic Health Record (EHR) chart review using a pre-determined chart-abstraction form for cases and controls. Chart review was conducted by two independent reviewers as a part of an internal validation process for accuracy. Specifically, we noted surgical history including tonsillectomy status as listed in peri-operative and anesthesia reports. Given the chart-review nature of this study, details about timing and indication for tonsillectomy were often not available. However, when date of tonsillectomy was available, this was noted and qualitatively described. Data abstraction for cases included esophageal cancer specific subtype. BE was defined as columnar epithelium with goblet cells. Prior diagnosis of dysplasia (non-dysplastic, low grade dysplasia, or high-grade dysplasia) was noted. Many patients were referred from outside hospitals and length of Barrett’s segment was missing for many patients and thus could not be included in the analysis. For both cases and controls, demographic factors as well as comorbidities including previous cancer history, and potentially relevant exposures including GERD, prior BE diagnosis, alcohol use, tobacco use, proton pump inhibitor (PPI) and aspirin use were all recorded [1]. Given previous work showing that quitting smoking is not associated with a strong reduction in risk for EAC [15], a combined variable of any tobacco use was used in the primary analysis. A similar method was adopted for alcohol use. Due to the association of obesity with BE and EAC, we calculated BMI from height and weight at the time of thoracic surgery from weight and height available in the electronic medical record. While weight was recorded for most patients, height was missing for about half of the patients. Research Electronic Data Capture (REDCap) software, a secure, web-based application designed to support data capture for research studies, was used to create the research database [16]. A de-identified version of this database was then used for data analysis.

### Statistical analysis

This retrospective cohort study was designed to detect the association of tonsillectomy with HGD/EAC with over 80% power to and 5% significance level. Our primary outcome measure was presented as the frequencies and proportions by case and control. The association was measured by odds ratios (ORs) with 95% confidence intervals (CIs) and generated by using univariate tests, and then verified by multivariate logistic models with the considerations of associated covariates. Differences in demographics, clinical risk factors and comorbidities between cases and controls were preliminarily identified using a Pearson χ^2^ test or t-test. Rates of GERD and history of tonsillectomy were reported as frequencies (percentage) or mean (standard deviation).

Due to the high frequency of missing BMI data, we applied a model-based imputation approach that used simulated values to address the influence of missing values on results. By fitting the multivariate logistic/regression models with non-missing data, missing values were imputed from the posterior predictive distribution of the parameters. For BMI classification, obesity was defined as a BMI of 30 or more [17, 18]. Missing values in BMI were simulated by considering age, gender, height, weight, and race indices as the predictors. For missing values of tobacco use, age, gender, and race indices, prior tobacco, and ETOH status were included. Similarly, missing values for alcohol use were simulated by age, gender, and race indices, historical ETOH, and the tobacco use status.

Univariate and multivariate logistic regression analysis was used to generate unadjusted or adjusted estimation of the ORs (both point and interval estimation), which detected the association between HGD/EAC and baselines or between tonsillectomy with selected HGD/EAC risk factors (age, gender, BMI, race, GERD, history of BE, ever using tobacco and ever using alcohol). Stepwise model selection and clinical were both taken into our considerations to illustrate these relationships.

Finally, we further evaluated the relationship between *HGD/EAC* and tonsillectomy *with* incorporating all known HGD/EAC risk factors in a multivariate logistic model. Different models with or without missing imputations had been considered allowing assessment of the potential impact of the missing values on results.

The models were developed using existing data and also after imputing missing data to determine odds ratios to describe the association between tonsillectomy and other variables on risk of HGD/EAC. Statistical analysis was performed using SAS software, version 9.4 (Cary, NC, USA). SAS and all other SAS Institute Inc. product or service names are registered trademarks or trademarks of SAS Institute Inc., Cary, NC, USA. Version 9.4 SAS Software, Copyright 2013.

## Results

### Demographic distribution and medical history of cases and controls

Given known racial and gender distribution of EAC, as expected, patients with HGD/EAC were more likely to be white and male. While we had initially filtered control subjects by age, some of the selected controls did not meet inclusion criteria after more detailed chart review, changing the demographics of the final control group. However, overall, no significant difference in age was detected between the two populations.

Due to the known natural history of EAC, in the unadjusted analysis, a history of BE, GERD and PPI use were all more common in cases versus controls, as reported in Table 1. Due to study design, rates of BE in the control group were very low. Rates of exposures to risk factors associated with HGD/EAC, such as tobacco and alcohol were high in both groups, but significantly higher among cases (Table 1). Prior rates of non-esophageal cancer were found to be higher in thoracic surgery control group than in the esophageal cancer case group. Similarly, frequencies of comorbid conditions such as lung disease, liver disease and kidney disease were higher in controls than in cases (Table 1). However, metabolic diseases including diabetes mellitus, history of myocardial infarction and cardiovascular disease were similar between groups; use of a statin medication was also similar between groups. BMI was also similar between both groups. 25% of cases versus 1% of control patients had a reported history of BE (Table 1).

**Table 1:** Baseline characteristics and medical history for HGD/EAC cases and thoracic surgery controls.

Characteristic	Case N = 452 (29%)	Control N = 1,102 (71%)	Overall N = 1,554	P-values to detect a difference
Age Mean (SD)	63 (10)	62 (6)	62 (7)	0.041
Male N (%)*	395 (87)	620 (56)	1,015 (65)	<.001
BMI Obesity N (%) 47% missing	47 (29)	206 (31)	253 (31)	0.564
Race- White N (%)*	424 (94)	885 (80)	1,309 (84)	<.001
GERD N (%)*	268 (59)	454 (41)	722 (46)	<.001
Proton Pump Inhibitor N (%)*	289 (64)	402 (37)	691 (44)	<.001
H2 blocker use N (%)*	16 (4)	78 (7)	7 (6)	0.008
ASA use N (%)*	81 (18)	365 (33)	446 (29)	<.001
NSAID use N (%)	18 (4)	63 (6)	81 (5)	0.163
Statin use N (%)	106 (23)	244 (22)	350 (23)	0.575
Barrett’s esophagus N (%)*	113 (25)	15 (1)	128 (8)	<.001
Peptic ulcer disease N (%)*	40 (9)	49 (4)	89 (6)	0.001
Previous History of cancer N (%)*	81 (18)	432 (39)	513 (33)	<.001
Diabetes mellitus N (%)	93 (21)	256 (23)	349 (22)	0.255
Myocardial infarction N (%)	49 (11)	102 (9)	151 (10)	0.338
Coronary artery disease N (%)	90 (20)	230 (21)	320 (21)	0.671
Lung disease N (%)*	110 (24)	656 (60)	766 (49)	<.001
Liver disease N (%)*	11 (2)	55 (5)	66 (4)	0.023
Kidney disease N (%)*	41 (9)	170 (15)	211 (14)	0.001
Ever Tobacco N (%)* 6% missing	338 (81)	711 (68)	1049 (72)	<.001
Ever Alcohol N (%)* 18% missing	232 (66)	356 (39)	588 (46)	<.001

* Factors significantly different between case versus control were identified based on Chi-square test or the estimated 95% confidence intervals (95% CIs) of means. Please note that the p-value for Age was lower than 0.05 but the 95% CI were overlapping (61.7–63.6) for case and (61.2–61.9) for controls, which did not imply significance.

### Distribution of dysplasia and esophageal cancer subtypes in the esophagectomy cohort

Distribution of the types of esophageal cancers within the esophagectomy database was consistent with the known higher rates of EAC compared with ESCC in the US; most of the esophageal cancers were of the adenocarcinoma subtype. The 81 ESCC cases in the esophagectomy database were not included in the primary case-control analysis. In the 452 cases of EAC/HGD from the esophagectomy cohort, only 113 (25%) had a prior known history of BE. As suggested by the literature [19], diagnosis of BE generally did not precede diagnosis of cancer. It is also important to note that based upon pathologic description, BE was not always detected on pathologic assessment of the cases at esophagectomy; of all the 452 HGD/EAC cases, 45.8% did not include a final pathologic description of BE. Of the 396 EAC cases, when the BE adjacent to the EAC was evaluated for the presence of dysplasia after esophagectomy, 52.3% of pathology reports suggested no BE was present, 20.7% had non-dysplastic BE, 4.0% had LGD, 15.6% had HGD and 7.1% had intramucosal adenocarcinoma. Within the cohort of 452 HGD/EAC cases, 264 tumors were at the GEJ. In the selected group of GEJ cancers, 91.3% did not have a known history of BE and 70.5% did not include a pathologic description of BE on the pathology report at the time of esophagectomy.

### Prior history of tonsillectomy was associated with increased risk for HGD/EAC

**Fig 1:**
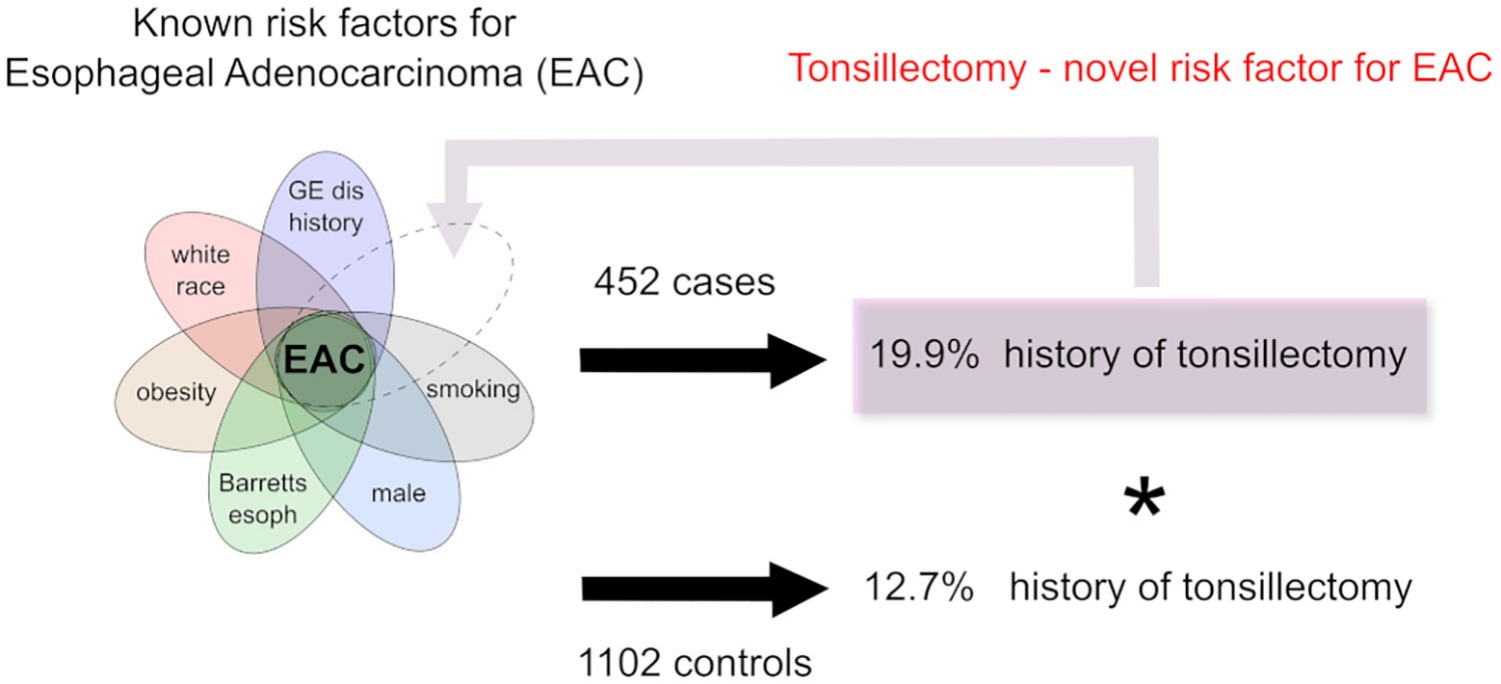
Illustration of our study showing the distribution of tonsillectomy in our cases and controls in relation to known risk factors for HGD/EAD.

As illustrated in Fig 1, of the HGD/EAC cases in our analysis, a total of 90 (19.9%) cases had a prior history of tonsillectomy compared to a total of 140 control group patients (12.7%) with a prior history of tonsillectomy (p-value 0.0003) (Fig 1). In a univariate analysis, patients with a prior history of tonsillectomy were more likely to have HGD / EAC compared to those with no prior history of tonsillectomy OR 1.7 (95% CI 1.3–2.3). This association persisted even after adjustments for covariates OR 1.8 (95% CI 1.2–2.7).

Due to the chart-review nature of the study, data in the medical record were incomplete regarding timing of tonsillectomy; surgical history often listed the procedure without additional information such as timing. A review of the 603 esophagectomy patients revealed that for seven patients, a qualitative description of timing of tonsillectomy was provided and in all these instances, tonsillectomy had been performed in childhood (n = 5), remotely or in the patient’s 20s (at least five decades before esophagectomy). For the 25 instances where a year of tonsillectomy was provided, 22 of the 25 were between the 1930s and 1970s (at least 30 years prior to the esophagectomy) and only 3 of the 25 were in the 2000s, closer to the time of esophagectomy.

Next, we evaluated the association between known HGD/EAC risk factors and tonsillectomy. White race, history of GERD and coronary artery disease (CAD) were all associated with a history of tonsillectomy (Table 2). In Table 3, we adjusted odds ratios using multivariate logistic regression with or without missing imputation. Despite the different models, the ORs were similar with consistent association of white race, reflux history and CAD with tonsillectomy (Table 3).

**Table 2:** Association between various known risk factors for EAC and prior tonsillectomy are presented here.

Characteristics	Levels	Tonsillectomy N = 230 (15%)	p-values	Odds Ratios (95% CI)
Age	Difference from non-Tonsillectomy Mean (SD)	0.4(7)	0.439	1.0 (0.99, 1.03)
Gender	Male	140 (17%)	0.125	0.8 (0.60, 1.07)
	Female	90 (14%)	-	ref
BMI	BMI Obesity	40 (16%)	0.610	1.1 (0.75, 1.71)
	BMI Normal to Overweight	82 (14%)	-	ref
	N/A	108 (15%)	0.954	1.1 (0.77, 1.51)
Race*	White	210 (16%)	0.002	2.2 (1.33, 3.48)
	Non-white	20 (8%)	-	ref
GERD*	Yes	128 (18%)	0.003	1.5 1.16, 2.04)
	No	102 (12%)	-	ref
Barrett’s esophagus	Yes	22 (17%)	0.428	1.2 (0.75, 1.97)
	No	208 (15%)	-	ref
Coronary artery disease*	Yes	31 (10%)	0.004	0.6 (0.37, 0.83)
	No	199 (16%)		ref
Ever tobacco	Yes	153 (15%)	0.705	1.0 (0.70, 1.32)
	No	62 (15%)	-	ref
	N/A	15 (16%)	0.805	1.1 (0.57, 1.95)
Ever alcohol	Yes	100 (17%)	0.081	1.3 (0.99, 1.83)
	No	91 (13%)	-	ref
	N/A	39 (14%)	0.655	1.1 (0.71, 1.59)

* Odds Ratios (ORs) describe the association of each risk factor with tonsillectomy.

* Factors significantly associated with Tonsillectomy were concluded based on the 95% Confidence Interval of ORs. White race, GERD, and CAD were associated with a history of tonsillectomy.

* We didn’t identify significant differences from the missing groups for BMI, tobacco, and alcohol.

**Table 3:** Adjusted analyses of relationship between known EAC risk factors with prior tonsillectomy.

*Characteristics*	Levels	ORs Unadjusted	ORs Adjusted^1^	ORs Adjusted^2^	ORs Adjusted^3^
Age	Continuous	1.0	1.0	1.0	1.0
Gender	Male (ref: Female)	0.8	0.8	0.7*	0.7*
BMI	Obesity (ref: Under)	1.1	1.1	1.2	1.2
	N/A (ref: Under)	1.1	-	1.1	-
Race	White (ref: Non-white)	2.2*	2.2*	2.1*	2.1*
GERD	Yes (ref: No)	1.5*	1.5*	1.5*	1.5*
Barrett’s esophagus	Yes (ref: No)	1.2	1.2	1.0	1.0
Coronary artery disease	Yes (ref: No)	0.6*	0.6*	0.6*	0.6*
Ever tobacco	Yes (ref: No)	1.0	1.0	1.0	1.0
	N/A (ref: No)	1.1	-	1.2	-
Ever alcohol	Yes (ref: No)	1.3	1.4*	1.4	1.4*
	N/A (ref: No)	1.1	-	1.0	-

* Unadjusted ORs, as well as ORs adjusted to account for missing BMI, tobacco use and alcohol use data are presented. OR estimates were calculated using patients without Tonsillectomy as the reference group. Columns represented include: 1) Unadjusted ORs, 2) Adjusted ORs calculated univariately after imputation, 3) Adjusted ORs calculated by multivariate logistic regression with all variables included, and 4) Adjusted ORs calculated by multivariate logistic regression with all variables included after imputation for missing data. Factors significantly associated with Tonsillectomy were concluded based on the 95% Confidence Interval of ORs.

Next, a multivariate logistic regression analysis was performed in order to evaluate the association between tonsillectomy and EAC/HGD. These results are presented in Table 4. Male sex, white race and PPI use were strongly associated with HGD/EAC as was ETOH use. As expected, having ever used tobacco was a significant risk factor for EAC/HGD with OR 1.5 (1.06–2.24), after imputation. Interestingly, ASA use was protective for HGD/EAC in this case-control analysis with OR 0.3 (95% CI 0.17–0.39). Importantly, variables were created to determine if there was any effect of the missing data for BMI and tobacco use; having missing BMI data (versus being underweight) was associated with EAC. Consequently, multivariate analysis was performed with and without missing BMI imputation and overall, the association between prior tonsillectomy and EAC/HGD persisted (Table 4).

**Table 4:** Multivariate logistic regression to evaluate associations between tonsillectomy, esophageal adenocarcinoma, and other covariates. Two ORs are presented for each variable: 1) Before imputation of missing values (BMI, tobacco, alcohol); 2 After imputation of missing values.

Effect	OR^1^	95% Wald CI	OR^2^	95% Wald CI
Tonsillectomy	1.8*	(1.16,2.74)	1.8*	(1.19, 2.74)
Age	1.0*	(1.01, 1.05)	1.0*	(1.01, 1.05)
Gender (Male /Female)	8.6*	(5.64, 13.16)	8.5*	(5.66, 12.89)
BMI (Obesity vs Under)	0.9	(0.56, 1.56)	1.2	(0.86, 1.65)
(NA vs Under)	2.7*	(1.91, 3.87)	-	-
White/Non-White	3.1*	(1.81, 5.29)	3.0*	(1.75, 5.00)
GERD	1.2	(0.80, 1.66)	1.1	(0.76, 1.55)
PPI use	2.6*	(1.84, 3.81)	2.6*	(1.79, 3.64)
H2 blocker use	0.4*	(0.19, 0.93)	0.5*	(0.22, 0.98)
ASA use	0.3*	(0.18, 0.42)	0.3*	(0.17, 0.39)
NSAID use	0.7	(0.31, 1.39)	0.6	(0.31, 1.26)
Statin use	1.4	(0.94, 2.18)	1.2	(0.81, 1.83)
History of BE	21.2*	(10.67, 42.06)	23.0*	(11.63, 45.65)
Peptic ulcer disease	1.2	(0.66, 2.28)	1.1	(0.62, 2.11)
Previous History of Cancer	0.3*	(0.18, 0.39)	0.3*	(0.18, 0.38)
Diabetes mellitus	1.0	(0.64, 1.46)	0.9	(0.61, 1.39)
Myocardial infarction	1.0	(0.50, 1.87)	1.1	(0.59, 2.17)
Coronary artery disease	1.0	(0.56, 1.64)	0.9	(0.56, 1.59)
Lung disease	0.1*	(0.10, 0.19)	0.1*	(0.10, 0.19)
Liver disease	0.5	(0.19, 1.11)	0.5	(0.19, 1.11)
Kidney disease	0.4*	(0.25, 0.68)	0.5*	(0.29, 0.77)
Ever tobacco (Yes/No)	1.4	(0.92, 2.10)	1.5*	(1.06, 2.24)
Ever tobacco (N/A/No)	0.9	(0.40, 1.86)	-	-
Ever alcohol (Yes/No)	2.7*	(1.83, 3.85)	2.0*	(1.49, 2.81)
Ever alcohol (N/A/No)	2.1*	(1.31, 3.53)	-	-

* OR estimates were calculated using thoracic surgery controls as reference group. Factors significantly associated with HGD/EAC were concluded based on the 95% Confidence Interval of ORs.

## Discussion

Our single center case-control study presents a new association between prior tonsillectomy and HGD/EAC, when compared with other thoracic surgery control patients. This represents an association that, to our knowledge, has never been reported before. Particular strengths of our study include our large cohort (452 cases and 1102 controls), surgical patient populations with similar data collection practices, and rigorous data analysis that accounted for missing data points and adjusted for several potential covariates. Based upon those strengths, the intriguing association between tonsillectomy and prior history of tonsillectomy merits further research. In particular, validation in population-based cohort would be an important next step. An additional secondary finding from this analysis was a negative association between aspirin use and HGD/EAC. Other recent studies have also noted the potentially protective effect of aspirin in combination with high-dose PPI in reducing all-cause mortality [20].

Tonsillectomy has been reported to have age-dependent associations with other malignancies, including various head and neck cancers [9, 21]. Fakhry et al in their study using data from the Denmark Cancer Registry, demonstrated that tonsillectomy increases the incidence of both tonsil and base of tongue cancers in patients over 60 years but may have a protective effect against tonsil cancers in patients under 60 years of age [9]. Zevallos et al, also showed that tonsillectomy performed before the age of 13 significantly increased the risk for cancer at the base of the tongue [21]. The rise of tonsillectomies can be traced back to the early 20^th^ century when it was common practice to perform tonsillectomy as treatment for throat infections. The procedure was considered relatively benign and effective. Indeed, between 1915 and 1960, tonsillectomy was the most popular surgical procedure in the US [8]. However, with the increased use of antibiotics in clinical practice in the mid 20^th^ century, a greater than 50% decline in tonsillectomies was reported between 1977 and 1989 [22]. Our findings suggest that that tonsillectomies may predispose patients to adult malignancies such as EAC many years later, correlating with the rise in EAC noted in the 1970s. Incidence of tonsillectomies has again risen over the past 35 years, although the most common indication for the procedure has shifted from recurrent infection to sleep disordered breathing [8]. The recent resurgence from its application as treatment for sleep disordered breathing may also be related to obesity, a factor we controlled for in this analysis.

**Fig 2:**
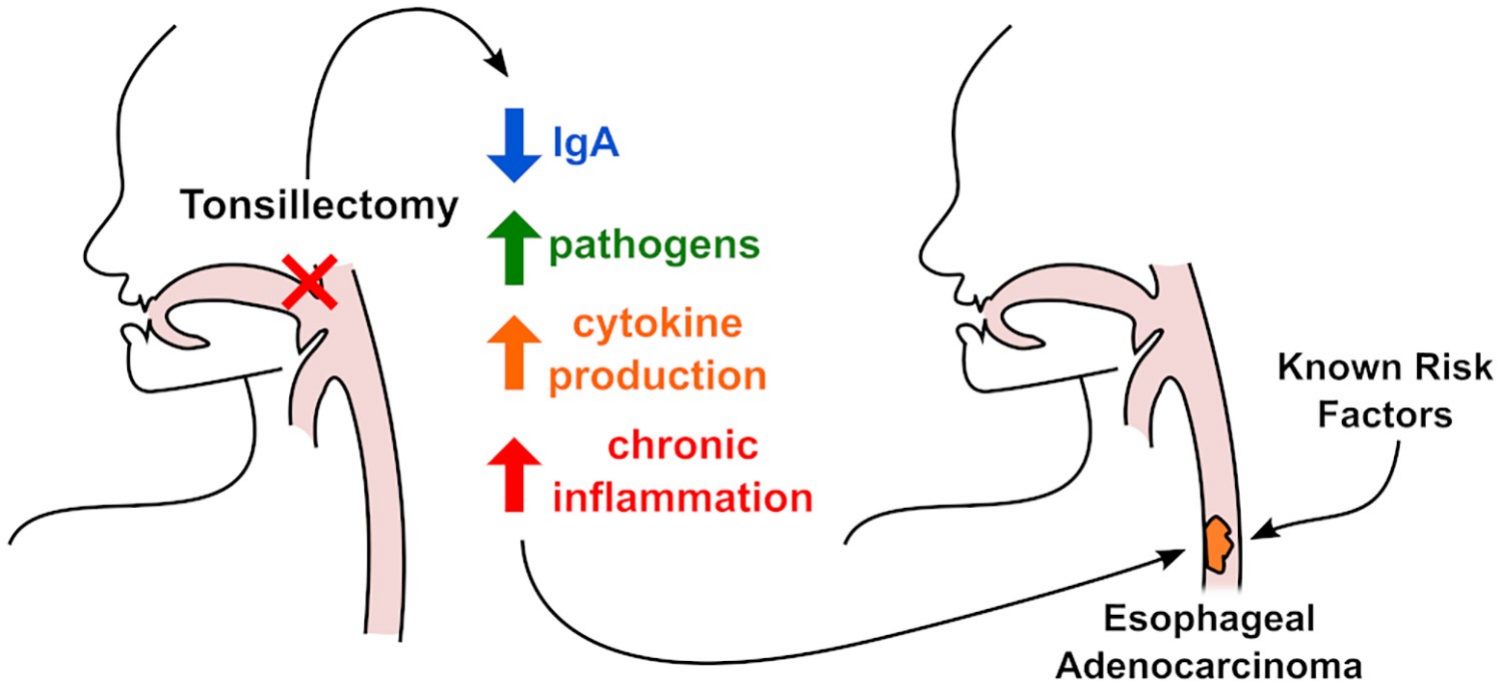
Illustration depicting possible association between tonsillectomy, inflammatory dysregulation, known EAC risk factors and development of EAC. Inflammatory dysregulation is proposed as an area for future investigation rather than as an established mechanism of disease.

A possible biologic explanation for the association between tonsillectomy and EAC relates to potential immune dysregulation associated with tonsillectomy that disrupts the esophageal and/or intestinal microbiome, increasing chronic exposure to harmful bacteria and uncontrolled stimulation of inflammatory pathways. Tonsils are known to be sentinel immune organs that protect the digestive tract from foreign pathogens and bacteria [23]. Tonsil dysfunction or removal may therefore impair the natural protective effect against incoming bacteria and/or disrupt the existing bacterial ecosystem. Studies have shown reduced levels of secretory immunloglobulins such as IgA after tonsillectomy. Mucosal IgA is needed as protection from mucosal pathogens; when IgA levels fall, this could contribute to increased proinflammatory signalling and risk for disease [24]. Other studies have shown that the esophageal microbiome is consistently altered in patients with GERD, BE and EAC, with increased prevalence of gram negative bacteria such as *Fusobacterium*, *Bacteroides*, *Veillonella*, and *Escerichia coli* and decreased prevalence of gram positive bacteria like *Streptococcus spp*. [25–32]. Although still unclear, it has been suggested that this shift in the microbiome from gram positive to more gram negative composition may trigger toll like receptors (TLR) such as TLR3 and TLR4 and increase expresson of downstream cytokines such as IL-8 and NFkB that promote inflammation associated carcinogenesis [27, 33]. Other factors such as the presence of *H*. *pylori* and increased microbial diversity, have shown a protective trend against EAC [25, 34–36]. Whether tonsilar dysfunction or removal increases esophageal neoplastic risk by impairing mucosal defense against harmful pathogens, directly modifying the esophageal/intestinal microbiome, or by stimulating chronic inflammation remains unknown and may be a promising area for future research. Fig 2 proposes potential mechanisms that could connect the risk HGD/EAC with tonsillectomy and while these are speculative, they present interesting avenues for more mechanistic research.

Other possible mechanisms, such as the presence of underlying genetic drivers, might be associated with both tonsillectomy and other conditions associated with EAC. Genome wide association studies have emphasized that GERD is a polygenic disease, and have shown an association between multiple GERD single nucleotide polymorphisms (SNPs) and the development of BE and EAC [37]. When we controlled for clinical GERD, prior history of tonsillectomy remained an independent risk factor for EAC. Given the complexity of both GERD and tonsillitis, we explored other underlying genetic factors that could be associated with GERD and tonsillectomy. Studies have implicated risk loci associated with childhood tonsillectomy in *SH2B4* and *HLA-B* genes [38]; these risk loci are also associated with increased risk for GERD [37]. While further exploration of associations between risk loci for tonsillectomy, GERD, BE and EAC are needed, another study suggested that functional polymorphisms in the immune-related gene for human beta defensin (*DEFB1)* were associated with increased susceptibility to adeno-tonsillar hypertrophy [39]. As illustrated with proposed mechanisms in Fig 2, it is possible that underlying genetic factors related to immune function could be associated with GERD, tonsillectomy and cancer development. Further population-based studies with genomic components as well as multi-center prospective trials may be required to better understand genetic links between tonsillectomy, immune function, and EAC.

Our study has several strengths, but it also has some limitations, many of which are inherent to the case-control, single-center, study design. First, although our rigorous imputation analysis accounts for missing BMI data, it is unlikely to completely eliminate the risk of confounding bias from obesity. Given the available data, it was not possible to control for prior remote antibiotic exposure, another potential confounder shown in a recent study from the UK to have a dose-dependent association with EAC [40]. Next, self-reported data presents a risk of recall bias, but previous studies have reported a sensitivity as high as 92.7% and specificity of 77.5% of tonsillectomy recall, and so we do not believe recall bias significantly impacted our results [21, 41]. Finally, our study was underpowered to determine associations between ESCC and tonsillectomy, as the relatively small number of ESCC cases limited our ability to draw conclusions on statistical significance. However, rates of tonsillectomy in ESCC cases were similar to controls. Despite these limitations, as efforts to prevent and treat EAC continue, and the incidence of tonsillectomy continues to rise, we believe our study offers an important novel association between tonsillectomy and EAC. However, we would caution clinicians to avoid making any conclusions about causality based upon these data. Indeed, tonsillectomy remains a relatively common procedure and esophageal cancer is still quite rare. The odds ratio associated with tonsillectomy is modest at best and should not imply causality. That said, the work presented here provides a basis for future studies that might test the validity of these findings on a population level and explore potential biological mechanisms underlying the association of tonsillectomy and HGD/EAC.

## Supporting information

S1 Data (XLS)

## Abbreviations

BE: Barrett’s Esophagus
EAC: Esophageal adenocarcinoma
GERD: Gastroesophageal Reflux Disease
HGD: High Grade Dysplasia
NSAID: Non-Steroidal Anti-Inflammatory Drug
PPI: Proton Pump Inhibitor
TLR: Toll Like Receptor
